# 

**Published:** 1852-03

**Authors:** 


### CONTENTS

OF NO. III.

---

### ORIGINAL COMMUNICATIONS.

ESSAYS AND CASES.

Art. I.—Miscellaneous Cases. By L. P. Yandell, M. D., Louisville, Ky. - - - - - - - 185
Art. II.—Vital Statistics of Marshall County, Ky. By George W. Irvin, M. D., of Benton, Ky. - - 196
Art. II.—Remarks on Fracture of the Cranium. By John Hardin, M. D., of Louisville, Ky. - - - 203

REVIEWS.

Art. IV.—Discourses delivered by appointment before the Cincinnati Medical Library Association, January 9th and 10th, 1852. By Daniel Drake, M. D. - - 220

SELECTIONS FROM FOREIGN AND HOME JOURNALS.

Amputation of the Lower Jaw, - - - - - - 245
Polypus of Larynx, - - - - - - - - - 248
Statistics of Fracture of the Cranium, - - - - - 249
On the Treatment of the Obstruction of the Bowels, - - 252
Practical Rules for the Suppression of Arterial Hemorrhage, - 255
On a Now and Simple Method for the Cure of Fistula, - 258
Poisoning with Oil of Bitter Almonds, - - - - - - 260
A Case of Ossification of the Placenta, occurring several times in the same Individual, - - - - - - - - 261
Paronychia an Epidemic, - - - - - - - - 264
Colica Pictonum from the medical employment of Acetate of Lead, - - - - - - - - - - - 266

Inhalation of Chloroform for the Relief of Phymosis, - - 267
Accidents caused by a very Small Dose of Santonine given to a Child, - - - - - - - - - - 268

ORIGINAL INTELLIGENCE.

University Law Suit, - - - - - - - - - 269
Registration Law of Kentucky, - - - - - - 270
Diminished Mortality from Consumption, - - - - - 272
Relative Mortality of the States, - - - - - - 273
Spiritual Writings, - - - - - - - - - - 273
Resignation of Prof. John Bell, - - - - - - 274
Medical Classes, - - - - - - - - - 274
Dr. Shields' Case of Consumption, - - - - - - 274
Circular on Lead Poison, - - - - - - - 275
Meteorological Table, - - - - - - - 276

